# A Midgut Duplication Cyst Lined by Respiratory Epithelium

**DOI:** 10.1155/2018/2678782

**Published:** 2018-03-12

**Authors:** Balaji Mahendran, Tim Bracey, Rajesh T. Kochupapy

**Affiliations:** ^1^Colorectal Surgery, Derriford Hospital, Plymouth PL6 8DH, UK; ^2^Histolopathology Department, Derriford Hospital, Plymouth PL6 8DH, UK

## Abstract

Duplication cysts are an uncommon finding. Majority of these cases are found in the region of the midgut, and many have been reported in literature. However, there has been only one previous case of a midgut duplication cyst lined by respiratory epithelium. This is a rare pathology, of which very little is known about. The pathophysiology of these cases is also difficult to explain. We aim to present a case of a midgut duplication cyst in a paediatric patient, who had other abnormalities as well. We also aim to offer a hypothesis for this case.

## 1. Introduction

Duplication cysts of the gastrointestinal tract are a rare anomaly and have been reported in literature mostly as incidental findings following investigations for symptomology that have not been explained by other pathology [1–5]. A duplication cyst is defined by a spherical hollow object lined by a mucous membrane, attached to any part of the gastrointestinal tract, and also having at least a single outer muscular layer [1, 6]. These are usually found in the ileum (70%) [7]. There has been some association demonstrated with other congenital abnormalities, specifically including vertebral body and genitourinary tract abnormalities [8, 9]. We present an interesting case of a midgut duplication cyst lined by respiratory epithelium, with a hypothesis based on the clinical presentation.

## 2. Case Report

**Figure 1:**
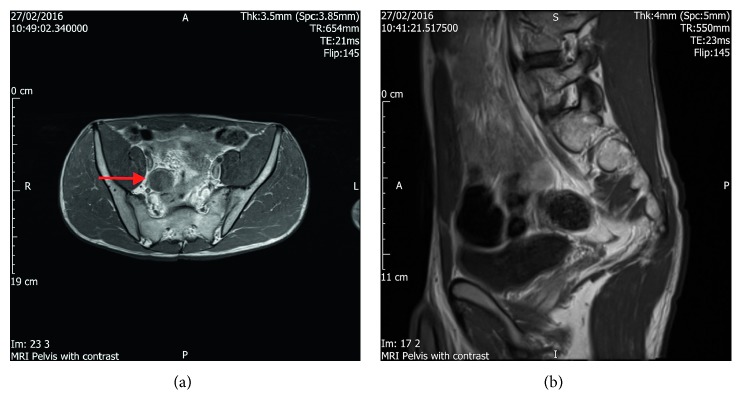
Initial MRI (without contrast) images.

A 16-year-old male presented from his general practitioner (GP) to the orthopaedic surgical department as he was complaining of back pain, and clinical examination had picked up a mild degree of scoliosis. The initial consultation with the orthopaedic surgeon had not revealed any abnormality; however, the patient was re-referred to the same department a year later, this time with marked scoliosis. A magnetic resonance image (MRI) was requested, which confirmed the presence of scoliosis but had also picked up an incidental cyst near the ileum, attached to the appendix (Figure 1).

**Figure 2:**
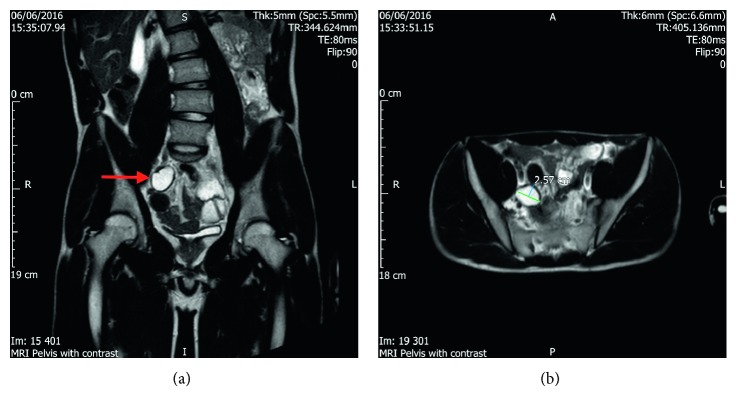
Subsequent MRI (with contrast) images.

A repeat MRI was performed 6 months later, which showed an unchanged cyst (Figure 2). Unfortunately, the first MRI was not done with contrast, and as such the patient had a third MRI with contrast, which characterized this as a possible duplication cyst. The patient was then found to have multiple neurofibromata, and a Lisch nodule as well.

**Figure 3:**
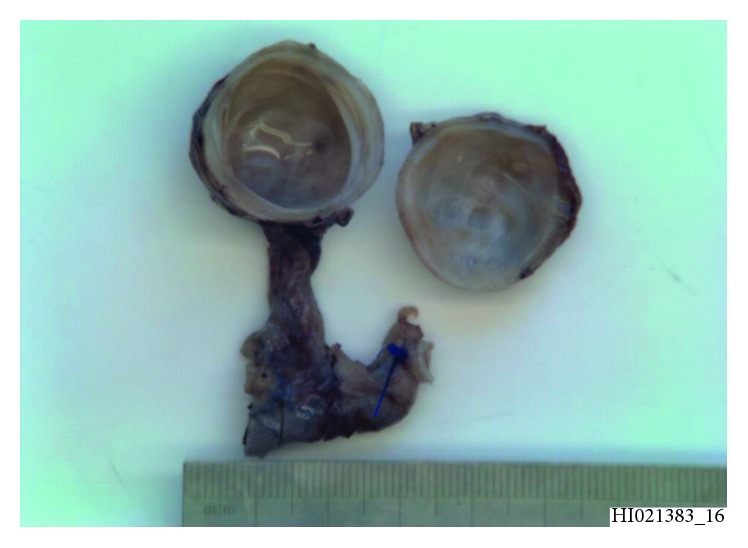
Macroscopic appearance of duplication cyst.

The cyst was excised as an elective procedure (Figure 3). An appendix measuring 55 mm in length by up to 11 mm in diameter was removed. There was also an attached rubbery nodule measuring 30 × 25 × 23 mm within the mesoappendix. The serosal surface of the appendix was unremarkable. The lumen and tip contained a faecolith. Slicing the nodule revealed a unilocular cyst measuring 20 mm in diameter containing gelatinous material.

**Figure 4:**
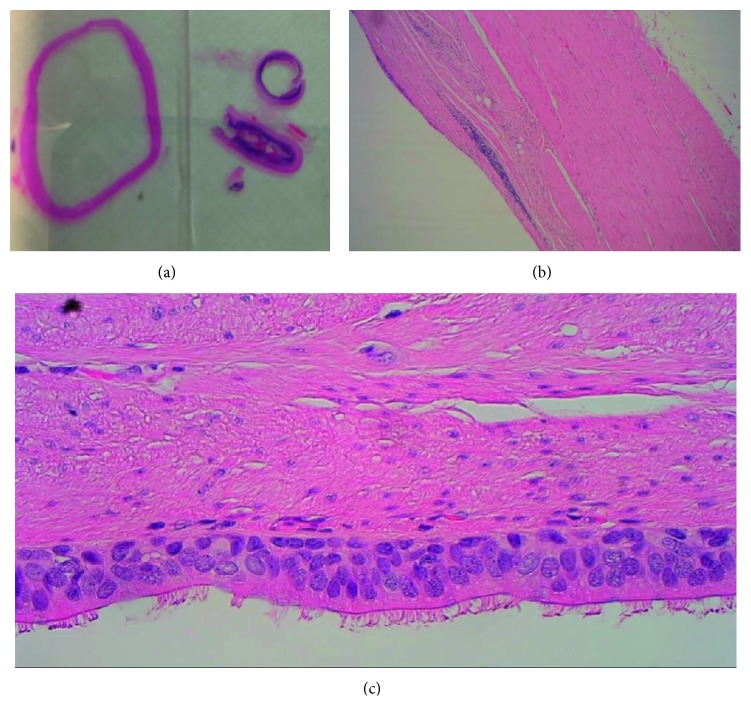
Histology of duplication cyst (stained with haemotoxylin and eosin).

Histology was performed, which showed a unilocular cyst, lined by pseudostratified ciliated columnar epithelium, with a discoloured wall showing no submucosa (Figure 4). The muscularis had an inner circular layer and an incomplete outer longitudinal layer. There were a few myenteric plexus-like fibres, which were mostly found in the adventitia. A few mural lymphoid aggregates were seen, with no other significant inflammatory infiltrate. The features were in keeping with a benign midgut duplication cyst, due to the presence of a muscular layer, completely encompassing the defined cyst.

## 3. Discussion

Duplication of the ileum, as discussed, are the most common cysts found in this population; however, an ileal duplication cyst lined by respiratory epithelium is rare [9]. There have been a few cases reported of foregut duplication cysts lined by respiratory epithelium [1, 2]; however, there is a relative paucity of literature on the presence of midgut duplication cysts being lined by respiratory epithelium, with one case being reported in Arizona, USA [5]. The complications of duplication cysts are that of any diverticulum of the gastrointestinal tract—including perforation, intussusception, bowel obstruction, and neoplastic change [5].

The embryological theories behind these cysts are plenty; however, most theories suggest some kind of anatomical relation between the location of the duplication cyst and the histopathological findings of these cysts. The split notochord theory proposes a neural tube traction mechanism as an explanation for the 15% of enteric duplications associated with vertebral defects. Specifically, an embryologic error may result in abnormal diverticularization of the GI endoderm through the developing notochord at 4 weeks' gestation. This explains neurenteric cysts, which are proposed to be impaired separation of the notochord from intestinal endoderm and formation of neurenteric bands that, with embryonal growth, produce traction diverticula. This is currently in favour and has been demonstrated experimentally in amphibian embryos [10].

In the embryogenesis of the foregut, a respiratory diverticulum appears at the ventral wall of the pharyngeal gut at 4 weeks. This is slowly divided into a ventral and dorsal part of the foregut, becoming the respiratory primordium and the upper gastrointestinal tract, respectively [10]. The oesophagus is initially lined by respiratory epithelium, eventually undergoing subsequent metaplasia and differentiating into squamous epithelium. Gastric duplication cysts lined by respiratory epithelium could be explained by the budding of the tracheal trifurcation in early embryos, and not undergoing metaplasia to subsequently form gastric mucosa.

This cyst was thought to be a duplication cyst due to the typical findings on histology. The fact that it was found completely in the mesoappendix, without any communication with the gastrointestinal tract, was also unexpected. This could possibly be due to the aforementioned abnormal diverticulisation of this cyst. However, the presence of respiratory epithelium in this cyst is one that is unusual, and might represent a developmental abnormality. The patient had genetic testing done to check for relevant syndromes; however, that was negative. Further case series will be beneficial in expanding our knowledge of this condition, especially with regard to the pathophysiology of the formation of a duplication cyst.
